# The association between systemic glucocorticoid therapy and the risk of infection in patients with rheumatoid arthritis: systematic review and meta-analyses

**DOI:** 10.1186/ar3453

**Published:** 2011-08-31

**Authors:** William G Dixon, Samy Suissa, Marie Hudson

**Affiliations:** 1 Arthritis Research UK Epidemiology Unit, Manchester Academic Health Science Centre, Stopford Building, The University of Manchester, Oxford Road, Manchester, M13 9PT, UK; 2 Centre For Clinical Epidemiology, Lady Davis Institute for Medical Research at the Jewish General Hospital, McGill University, 3755 Côte Ste-Catherine Road, Montreal, Quebec H3T 1E2, Canada

## Abstract

**Introduction:**

Infection is a major cause of morbidity and mortality in patients with rheumatoid arthritis (RA). The objective of this study was to perform a systematic review and meta-analysis of the effect of glucocorticoid (GC) therapy on the risk of infection in patients with RA.

**Methods:**

A systematic review was conducted by using MEDLINE, EMBASE, CINAHL, and the Cochrane Central Register of Controlled Trials database to January 2010 to identify studies among populations of patients with RA that reported a comparison of infection incidence between patients treated with GC therapy and patients not exposed to GC therapy.

**Results:**

In total, 21 randomised controlled trials (RCTs) and 42 observational studies were included. In the RCTs, GC therapy was not associated with a risk of infection (relative risk (RR), 0.97 (95% CI, 0.69, 1.36)). Small numbers of events in the RCTs meant that a clinically important increased or decreased risk could not be ruled out. The observational studies generated a RR of 1.67 (1.49, 1.87), although significant heterogeneity was present. The increased risk (and heterogeneity) persisted when analyses were stratified by varying definitions of exposure, outcome, and adjustment for confounders. A positive dose-response effect was seen.

**Conclusions:**

Whereas observational studies suggested an increased risk of infection with GC therapy, RCTs suggested no increased risk. Inconsistent reporting of safety outcomes in the RCTs, as well as marked heterogeneity, probable residual confounding, and publication bias in the observational studies, limits the opportunity for a definitive conclusion. Clinicians should remain vigilant for infection in patients with RA treated with GC therapy.

## Introduction

Infection is a major cause of morbidity and mortality in patients with rheumatoid arthritis (RA) [1,2]. The increased incidence has been attributed to the disease itself, associated factors such as smoking and immunosuppressive therapy, or a combination of these. Glucocorticoid (GC) therapy, still widely used in the treatment of RA [3], is thought to be associated with an increased infection risk as well as other well-established adverse effects [4]. GCs are known to impair phagocyte function and suppress cell-mediated immunity, thereby plausibly increasing the risk of infection [5]. However, the extent to which GC therapy contributes to the observed increased risk in RA is not clear.

Surprisingly, despite six decades of clinical experience [6], no good summary estimates of infectious risk associated with GC therapy in RA populations exist. Systematic reviews have been performed to address the *efficacy *of GC therapy [7], as well as multiple safety outcomes from RCTs in RA populations [8,9]. Reviews of safety issues from observational studies tend to be narrative (rather than systematic) reviews, despite the recognition that observational data must complement RCT data when assessing the harms of drug treatments [10]. No systematic reviews or meta-analyses exist that focus on the infection risk associated with GC therapy by combining evidence from RCTs and observational studies.

Our primary aim was to perform a systematic literature review and meta-analysis (where appropriate) of RCTs and observational studies to assess the association between systemic GC therapy and the risk of infection in patients with RA, compared with patients with RA not exposed to GC therapy. Secondary aims were to examine the influence of study design, definition of GC exposure, and type of infection.

## Materials and methods

### Search strategy

A search was conducted in MEDLINE, EMBASE, CINAHL, and the Cochrane Central Register of Controlled Trials (Clinical Trials; CENTRAL) database to January 2010 to identify studies among populations of patients with RA that reported a comparison of infection incidence between patients treated with GC therapy and patients not exposed to GC therapy.

Published studies were identified by using separate search strategies for RCTs and observational studies. The full search strategy can be found in Additional file 1. In brief, all GC RCTs for RA were sought. Observational studies were identified by using the broad keyword areas of "rheumatoid arthritis," "infection," and "antirheumatic therapy," limiting the search to epidemiologic studies. An initial search strategy of "GC therapy," as opposed to "antirheumatic therapy," missed many studies in which the association between GCs and infections was reported, but in which GC therapy was not included in the title, abstract, or as a key word. Exposure was limited to systemic GC therapy: studies that reported only intra-articular steroids were excluded. We considered only articles published in English because of the need to screen large numbers of publications by using the complete manuscript. Hand searching of reference lists from obtained articles and selected review articles also was performed. Abstract-only publications and unpublished studies were not considered. No authors were contacted for additional information.

### Study selection

The first selection, based on title and abstract, was done by one reviewer (WGD). Studies conducted exclusively in non-RA populations were excluded. Studies with designs other than RCTs, case-control, or cohort studies were excluded at this stage, as were studies of nonsystemic GC therapy. RCTs that did not randomize GC therapy were excluded. Case-control studies defined by any outcome except infection also were excluded. The full manuscripts of all remaining articles were obtained. Any uncertainty during initial screening led to retention of the article for eligibility assessment.

Eligibility assessment was then performed independently by two reviewers (WGD and MH), applying the following final study-inclusion criteria. For RCTs: (1) study population of patients with RA or undifferentiated inflammatory polyarthritis, (2) exposure to systemic GC therapy (that is, excluding intra-articular and tendon-sheath injections) in one arm and nonexposure in a further study arm (that is, in which the only major difference between the arms was the use of GC), and (3) reporting of infection numbers or rates in the two relevant study arms. If studies reported additional arms examining the effect of an alternative active treatment, data were analyzed only for the arms comparing GC therapy with no-GC exposure. If studies were explicit in describing the methods by which they captured infection, nonreporting of infection within the results was assumed to represent no infections in either group. Absent reporting of infection that was in any way ambiguous led to exclusion of the study. Studies that reported only adverse events leading to drug discontinuation were included, although grouped separately. For observational studies: (1) assessment of infection risk in a population (or subpopulation) of patients with RA or undifferentiated inflammatory polyarthritis, (2) use of a cohort or case-control design to conduct data analysis, and (3) provision of a relative-risk or rate-ratio estimate for the association between systemic GC therapy and infection with a corresponding 95% confidence interval (or sufficient data to calculate this) were required. These criteria allowed inclusion of open-label extension studies if they analyzed infection risk with GC therapy compared with no-GC therapy. *Helicobacter pylori *infection was excluded. Disagreements were resolved by discussion.

### Data extraction and meta-analysis

Data on the number of infections or the estimated relative risks were extracted by one reviewer (WGD), along with characteristics of the studies. Extracted data were cross-checked against notes made by both reviewers during the eligibility assessment, with resolution by discussion in the few instances of disagreement. Information on categorization of GC exposure and types of infection was collected.

Meta-analysis was conducted for RCTs and observational studies separately. RCT meta-analysis was performed initially including all studies, followed by a series of *a priori *sensitivity analyses. In the main analysis, all GC-treated arms were combined. Because of the low number of events and the sensitivity of the default weighting (the inverse of the variance of the logarithm of the odds ratio) to the definition of infection (for example, serious or not serious), alternative weighting was performed by number of patients, then by estimated person years of follow-up. To avoid excluding studies in which zero events were found in both arms, a sensitivity analysis was performed after adding 0.5 to all cells of the 2 × 2 table. Additional sensitivity analyses included limiting studies to GC doses of < 10 mg prednisolone equivalent (PEQ), limiting outcomes to serious infections, and excluding studies reporting only events leading to study withdrawal. If studies reported more than one type of infection, sensitivity analyses were performed to examine the influence of using alternative definitions. Different analysis methods were considered, given the statistical challenge of rare events [11], including the Mantel-Haenszel odds ratio (with and without zero-cell correction), inverse variance, and weighting by study size.

A meta-analysis of all observational studies was performed, stratified by study design (cohort and case control). If several strata of exposure (for example, 0 to 5, 5 to 10, and > 10 mg PEQ) were presented in the absence of an overall effect measure, one reported category was selected for the meta-analysis. If three categories were reported, the middle category was chosen. If only two categories were reported, the category with the larger number of patients or person time was selected. Random-effects models were used to account for between-study heterogeneity by using the DerSimonian and Laird method [12]. Similarity between the risk ratio and the odds ratio was assumed because infectious events were considered rare. Again, several *a priori *sensitivity analyses were conducted. With respect to exposure, dose-specific analyses were performed, as well as limiting analysis to studies considering only current GC exposure. Adjusted and unadjusted analyses were considered separately, as well as exploration of the impact of different components of multivariate adjustment (age and sex, disease severity, disease duration, comorbidity, and other RA therapies). Several specific outcomes were considered separately, including all-site serious infections, lower-respiratory-tract infections, tuberculosis, herpes zoster, and postoperative infections. In response to reviewers' comments, we also performed a sensitivity analysis of serious infections reported in prospective studies.

Funnel plots were created to examine the potential for small study effects [13]. Statistical heterogeneity was assessed by using the Cochrane I^2 ^statistic [14], in which I^2 ^> 50% represents substantial heterogeneity. All analysis was conducted by using Stata/SE version 11.

## Results

**Figure 1:**
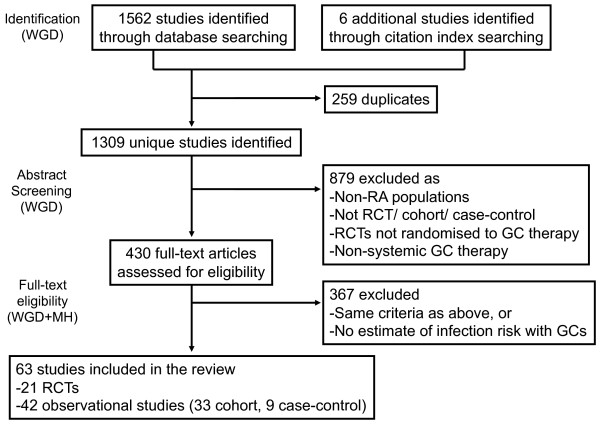
**Flow chart demonstrating study selection**. GC, glucocorticoid; RA, rheumatoid arthritis; RCT, randomized controlled trial.

The 1,568 records were identified through parallel database searching (Figure 1). The results were loaded into an electronic bibliographic management system (EndNote). After removal of duplicates, 1,309 studies were identified and screened by one reviewer (WGD). The 430 full-text articles were then assessed for eligibility by two reviewers (WGD and MH). The 21 RCTs [15-35] and 42 observational studies [36-77] (33 cohort, nine case-control) were included in the analysis. Details of the studies are described in Tables 1 and AF2 (Additional file 2).

**Table 1:** Summary of GC RCTs reporting infection outcomes

First author and year	Country	Setting/Population	Arms of RCT (*n*)	Duration of study	Type of outcome	Result
Boers, 1997 [15]	The Netherlands and Belgium	155 early RA patients from 8 centers	Combination therapy - step-down prednisolone from 60 mg, step-down MTX and SSZ (76) vs SSZ monotherapy (79)	28 weeks	Infections treated as outpatient	12 infections in combination arm, 6 in SSZ monotherapy arm
Chamberlain, 1976 [16]	UK	49 adult RA patients from single center	5 mg prednisolone (20) vs 3 mg prednisolone (10) vs 0 mg prednisolone (19) Allowed concomitant gold	2- 3.5 years	n/a	No infections
Choy, 2005 [17]	UK	91 patients with established RA with incomplete response to DMARDs. Multicenter study	Monthly 120-mg intramuscular depomedrone (48) vs placebo (43) Allowed usual DMARDs	2 years	n/a	No infections either arm
Choy, 2008 [18]	UK	467 patients within 2 years of diagnosis from 42 centers	MTX (117) MTX + cyclosporin (119) MTX + step-down prednisolone (115) MTX + cyclosporin + prednisolone (116)	2 years	a) All-site serious infections b) Respiratory tract infections	a) 7, 3, 4, and 2 serious infections in the four respective arms b) 54, 51, 49, and 55 respiratory tract infections in the four respective arms
Durez, 2007 [19]	Belgium	44 patients with early RA	MTX monotherapy (14) MTX + 1 g iv methylprednisolone^a ^(15) MTX + infliximab^a ^(15) Infusions weeks 0, 2, 6; then 8 weekly	46 weeks	a) Serious infection b) 'benign' infection	a) No serious infections in any arm b) 14, 12, and 12 benign infections in the three arms, respectively
Durez, 2004 [20]	Belgium	27 patients with active RA despite MTX	MTX + 1 g iv MP week 0 (15) MTX + infliximab weeks 0, 2, and 6 (12)	14 weeks	Serious infections	None in either arm
Gerlag, 2004 [21]	The Netherlands	21 patients with active RA despite DMARDs	60 mg prednisolone week 1, then 40 mg prednisolone week 2 (10) Placebo (11)	2 weeks	n/a	1 skin infection in placebo arm only
Heytman, 1994 [22]	Australia	60 patients with active RA previously treated with NSAIDs	Gold plus either 1 g iv methylprednisolone weeks 0, 4, and 8 (30) or placebo (30)	24 weeks	All patient-reported side effects	No infections reported
Jasani, 1968 [23]	UK	9 patients with erosive RA	4 × 1-week crossover study of ibuprofen 750 mg, aspirin 5 g, prednisolone 15 mg, and lactose as placebo	4 weeks	n/a	No infections reported
Kirwan, 2004 [24]	Belgium, Sweden, UK	143 patients with active RA	Budesonide, 3 mg (37), budesonide, 9 mg (36), prednisolone, 7.5 mg (39), placebo (31)	12 weeks	a) Respiratory infections b) Viral infections	a) 7, 4, 6, and 1 respiratory infections in the 4 groups, respectively. b) 4, 1, 0, and 0 viral infections in the four groups, respectively
Liebling, 1981 [25]	US	10 patients with active RA	Crossover trial of monthly 1-g iv methylprednisolone vs placebo	12 months (6 months per arm)	n/a	4 infections on placebo, 2 on GC
Murthy, 1978 [26]	UK	24 patients with > 30 minutes morning stiffness	Indomethacin, 25 mg × 4 (12), prednisolone, 5 mg (12)	2 weeks	n/a	No infections reported
Sheldon, 2003 [27]	UK	26 patients with active RA	Budesonide (14) or placebo (12) plus usual DMARDs	4 weeks	n/a	2 cases of influenza (one from each group).
Van Everdingen, 2002 [28]	The Netherlands	81 patients with active, previously untreated RA	10-mg prednisolone (40), placebo (41)	2 years	Data reported on infections treated with antibiotics	17 infections in 40 patients in GC arm, 22 infections in 41 patients in placebo arm
Wassenberg, 2005 [29]	Germany/Austria/Switzerland	192 patients with active RA, disease duration < 2 years	Gold or MTX plus either 5 mg prednisolone (93) or placebo (96)	2 years	All adverse events collected, reported only if occurred in 3 or more patients	Total 4/93 and 3/96 (Bronchitis in 3/93 prednisolone group, 0/96 placebo group. Influenza in 1/93 prednisolone group, 3/96 placebo)
Williams, 1982 [30]	UK	20 patients with active RA	1-g iv methylpredisonolone (10) or placebo (10)	6 weeks	"Serious side effects"	None reported
Wong, 1990 [31]	Australia	40 patients with active RA previously treated with NSAIDs	Gold plus either three pulses of 1 g intravenous methylprednisolone weeks 0, 4, + 8 (20) or placebo (20)	24 weeks	Patients interviewed for all possible side effects	1 injection-site infection in placebo group
Capell, 2004 [32]	UK	167 patients with active RA on no DMARD therapy	SSZ plus either 7 mg prednisolone (84) or placebo (83)	2 years	Withdrawals due to side effects	No discontinuations due to infection in either group
Svensson, 2005 [33]	Sweden	250 patients with active disease on DMARD therapy	DMARD + prednisolone, 7.5 mg (119), DMARD alone, open, no placebo (131)	2 years	Adverse events leading to withdrawal	1 abscess in non-prednisolone group. No infections leading to discontinuation in prednisolone group
Van der Veen, 1993 [34]	The Netherlands	30 patients with active RA	Oral MTX plus either placebo (10) or 100 mg oral prednisolone days 1, 3, and 5 (10) or 1 g iv MP days 1, 3, and 5 (10)	1 year	Adverse events leading to discontinuation of *MTX*	1 pneumonia in placebo group (at week 12)
van Schaardenburg, 1995 [35]	The Netherlands	56 patients with active RA aged > 60 previously treated with NSAIDs	Chloroquine, 100 mg/day (28) (rescue with gold, then SSZ allowed) vs prednisolone 15 mg/day, tapered after 1 month (28)	2 years	Withdrawal due to adverse advents	No discontinuations due to infections in either group

DMARD, disease-modifying antirheumatic drug; iv, intravenous; ivMP, intravenous methylprednisolone; MTX, methotrexate; NSAIDs: nonsteroidal anti-inflammatory drugs; RA, rheumatoid arthritis; SSZ, sulfasalazine. ^a^Infusions weeks 0, 2, 6; then 8 weekly.

There were 1,963 patients included in the 21 RCTs, and 526,629, in the 42 observational studies. The mean study duration was 41 weeks for the RCTs, and the median follow-up time was 1.93 person years per patient for the 30 observational cohort studies for which follow-up time was available.

### Main results

#### RCTs

**Figure 2:**
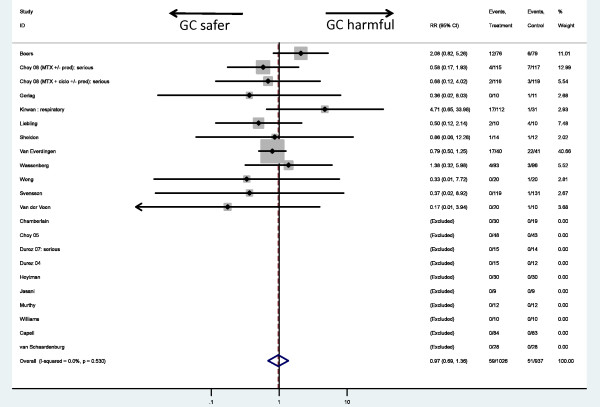
**Meta-analysis of infection risk in randomized controlled trials of systemic glucocorticoid therapy**.

In 1,026 GC-treated patients, 59 (5.8%) infections were found compared with 51 infections in 937 (5.4%) non-GC patients. Ten of 21 studies had no reported infections in either arm, and four further studies had no infections in one of the two arms. The estimated relative risk of infection associated with GC therapy was 0.97 (0.69, 1.36) (Figure 2). No evidence of statistical heterogeneity was present among the included trials (I^2 ^= 0.0).

#### Observational studies

**Figure 3:**
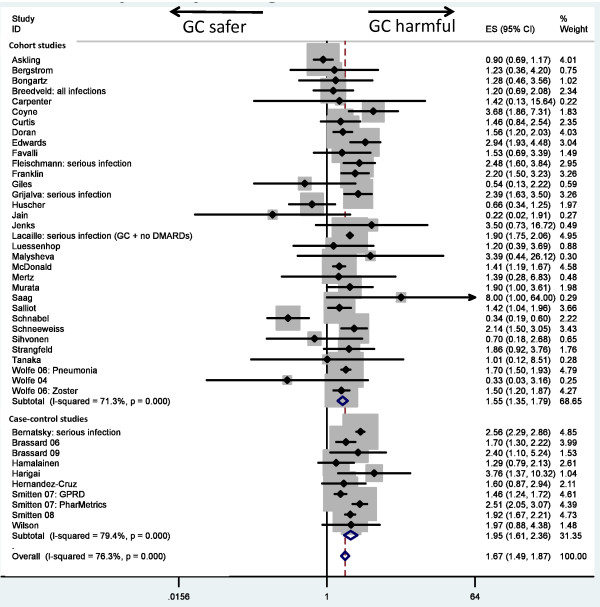
**Meta-analysis of infection risk in observational studies, stratified by study design (1, cohort; 2, case-control)**.

Systemic GC therapy was associated with an increased risk of infections in observational studies (RR, 1.67 (1.49, 1.87)). Risk estimates differed by study design, with cohort studies generating an RR of 1.55 (1.35, 1.79) and case-control studies, 1.95 (1.61, 2.36) (Table 2; Figure 3). However, evidence was noted of substantial statistical heterogeneity (I^2 ^= 76% for observational studies overall, 71% for cohort studies, and 79% for case-control studies).

**Table 2:** Study design factors within observational studies and their influence on relative risk of infection associated with glucocorticoid therapy

	Number of studies	Mean RR	I^2 ^statistic	Ratio of RR
Study design				
Cohort	33	1.55 (1.35, 1.79)	71.3%	1.00 (referent)
Case-control	9	1.95 (1.61, 2.36)	79.4%	1.26
Definition of exposure				
Baseline	5	1.46 (0.87, 2.45)	79.7%	1.00 (referent)
Current (within 3/12)	22	1.70 (1.47, 1.97)	58.9%	1.16
Recent (within 6/12)	7	1.56 (1.24, 1.96)	79.5%	1.07
Ever	2	1.80 (1.29, 2.51)	52.5%	1.23
Unclear	6	2.35 (1.27, 4.36)	36.5%	1.61
Adjusted for age and sex				
No	22	1.32 (0.97, 1.80)	67.6%	1.00 (referent)
Yes	19	1.78 (1.58, 2.01)	82.3%	1.35
Adjusted for disease severity				
No	24	1.41 (1.14, 1.75)	71.3%	1.00 (referent)
Adjusted for surrogate	10	1.98 (1.68, 2.34)	78.5%	1.40
Adjusted for direct measurement	6	1.52 (1.17, 1.97)	77.0%	1.08
Adjusted for disease duration				
No	33	1.63 (1.41, 1.89)	76.8%	1.00(referent)
Yes	6	1.55 (1.20, 2.01)	83.5%	0.95
Adjusted for comorbidity				
No	22	1.30 (0.97, 1.74)	64.2%	1.00 (referent)
Yes	17	1.74 (1.55, 1.96)	75.1%	1.34
Adjusted for other RA therapies				
No	22	1.28 (0.98, 1.67)	61.1%	1.00 (referent)
Yes	18	1.84 (1.62, 2.08)	82.8%	1.44

RR, relative risk.

### Sensitivity analyses

#### RCTs

Sensitivity analyses using alternative weighting, different statistical methods of dealing with low event numbers, limiting to studies with a placebo rather than active comparator, and limiting to doses < 10 mg PEQ led to no major change in the results (Additional file 3). Too few studies reported exclusively serious infections, and too few events in those studies, warranted a robust meta-analysis [18-20]. Studies considered to report predominantly nonserious infection generated an RR of 1.05 (0.89, 1.24). One study included methotrexate in addition to GC therapy in the treatment arm (15). Exclusion of this study generated an RR of 0.83 (0.57, 1.21).

#### Observational studies

Stratification by dose category showed a positive dose-response effect. Studies with average doses of < 5 mg PEQ generated an RR 1.37 (1.18, 1.58) compared with an RR of 1.93 (1.67, 2.23) for 5- to 10-mg PEQ. Only one study reported an RR for doses between 10 and 20 mg PEQ (RR, 2.97 (1.89, 4.67)) [68]. Limiting analyses to dose categories above a certain threshold also led to a dose response: RR, 2.46 (2.08, 2.92) for dose categories > 5 mg PEQ, RR 2.97 (2.39, 3.69) for dose categories > 10 mg PEQ, and RR 4.30 (3.16, 5.84) for dose categories > 20 mg PEQ. Doses of < 10 mg PEQ had a pooled estimate of 1.61 (1.42, 1.84), higher than the risk for studies of dosages < 5 mg PEQ.

Adjustment for age and sex led to an RR of 1.78 (1.58, 2.01) compared with no adjustment (RR 1.32 (0.97, 1.80)) (Table 2). Adjustment for direct measures of disease severity did not lead to much change in the risk estimates when compared with estimates not adjusted for direct measures of disease severity. Disease duration also had little impact on the RR. Adjustment for co-morbidity and for other RA therapies (disease-modifying antirheumatic drugs (DMARDs) and/or biologics) led to estimates ~40% higher than the unadjusted estimates. Limiting analysis to studies defining GC exposure as "current use" generated an RR of 1.70 (1.47, 1.97) (Table 2).

GC therapy was associated with an increased risk of all-site serious infection (RR, 1.89 (1.60, 2.24)), lower-respiratory-tract infections (RR, 2.10 (1.52, 2.91)), tuberculosis (RR, 1.74 (1.09, 2.76)), herpes zoster (RR, 1.74 (1.28, 2.36)) and, to a lesser extent, postoperative infections (RR, 1.38 (1.02, 1.86)). The risk of serious infections persisted when analysis was restricted to prospective studies (RR, 1.70 (1.14, 2.55)). Even with stratification by outcome, notable statistical heterogeneity remained across outcomes (I^2 ^= 82%, 51%, 28%, 86% and 0, respectively).

### Publication bias

The funnel plot of RCTs (Figure 3a) was roughly symmetrical, with all studies falling within the 95% CI. The funnel plot for observational studies was less symmetrical and had more outliers (Figure 3b). The Egger test for publication bias was nonsignificant for both the RCTs (*P *= 0.936) and observational studies (*P *= 0.174 for cohort studies and *P *= 0.576 for case-control studies).

## Discussion

RCTs and observational studies generated different estimates of infection risk associated with GC therapy. The RCT meta-analysis suggested a null association between GC therapy and infection risk (RR, 0.97 (0.69, 1.36)). The confidence interval included both clinically meaningful increased risks (up to 35% increase) and decreased risks (up to a 30% reduction), making the result inconclusive. The observational studies provided an overall RR of 1.67 (1.49, 1.87), suggesting a significant, clinically important increased risk. However, significant heterogeneity was found within the studies. Even after performing multiple sensitivity analyses around exposure definition, outcome, and adjustment for confounders, marked heterogeneity remained a problem. Nonetheless, most analyses of observational studies reported an increased risk of infection, which conflicts with the result of the RCTs. The dose of GC therapy varied both within and between RCTs and observational studies and may contribute to our observed result. However, we were able to perform meta-analyses within both study designs to investigate the risk associated with daily doses ≤ 10 mg PEQ. The differential results between study designs remained. Although it is not yet clear to what extent the risk of infection is influenced by historic (or cumulative) GC therapy, patients in the observational studies are likely to have had longer cumulative exposure than are patients within the short-duration RCTs. This difference may go some way to explaining the apparent discrepancy in the results from the two study designs.

Both study designs had major limitations when addressing infection risk. The big challenges in RCTs were poor reporting of methods and results and the statistical challenge of rare outcomes. For observational studies, heterogeneity, lack of detailed reporting, confounding, and bias (in particular publication bias) were particularly problematic. Other factors affecting the results and interpretation included variability of sampling frame, inclusion and exclusion criteria, definition of comparison groups, and time-varying GC exposure.

### Reporting of methods and results in RCTs

GC exposure was usually well defined within RCTs. On occasions, additional GC therapy was allowed at the discretion of the treating physician, and this was rarely quantified. In contrast, safety outcomes from RCTs lacked any standardized reporting of methods or results. Methods sections at times omitted any mention of safety assessment [30,78] or were too vague to be helpful (for example, "records of ... adverse reactions... were kept") [79]. In the results sections, selective reporting was problematic and included reporting of only pre-selected events (for example, fractures and ophthalmologic complications [80]), events known to be associated with GC therapy [17], events occurring in more than two patients [29], or events leading to withdrawal). Reporting only events with a frequency beyond a certain threshold would miss rare events, potentially imbalanced across multiple studies. Withdrawal studies (in which reporting was complete) provided measures of relative risk that could be included in the analysis. It is important that exclusion of these studies in a sensitivity analysis did not change the overall results. Vague reporting was also common. Phrases such as "no meaningful toxicities were reported by the participants in either group" [81] or "the proportion of patients who reported adverse reactions [did not] differ between groups according to type of treatment" [79] did not provide sufficient information on infections to warrant inclusion. Reporting of symptoms rather than diagnoses meant we had to decide subjectively (but independently) whether infections were present. We sought to include studies with an infection incidence of zero, only if this was explicit or could be confidently inferred. Although this was ambiguous at times, the use of two independent reviewers made study selection more robust.

Reporting of adverse drug reactions or side effects (with assumed causality) rather than all adverse events (in which causality is not assumed) was common. For a common event such as infection, causality is difficult to establish. Recent guidelines advise "terms that do not imply causality (such as 'adverse events') should be the default term to describe harms, unless causality is reasonably certain" [82].

Nonstandardized reporting in RCTs was a major problem in collating information. Different definitions of infection meant that summary risk estimates were averaged across different outcomes. We attempted to perform sensitivity analyses limited to serious or nonserious infections but were limited by low numbers. Underreporting of nonserious infections was likely: nonserious respiratory infections account for 300 to 400 general practice consultations annually per 1,000 registered patients in the United Kingdom [83]. Applying these rates to the RCTs, for example in the 2-year study of 192 patients by Wassenberg [29], we might expect > 100 nonserious infections. The reported number of infections was only seven.

### Rare events in RCTs

Much debate has occurred about the analytic and methodologic challenges of conducting meta-analyses to examine rare outcomes [11]. We used a variety of techniques including the Mantel-Haenszel odds ratio (with and without zero-cell correction), inverse variance, and weighting by study size to explore sensitivity to change. Although all methods failed to show a definite harmful or protective effect of GC therapy, all analyses included clinically important harms and benefits within the confidence intervals. GC therapy might be associated with a ≤ 35% increased risk of infection, or a 30% reduction. Although GCs are widely thought to increase the risk of infection, it is plausible that they might decrease the risk at these lower doses by controlling disease severity. The broad confidence intervals that span regions of clinically important effects in both directions are a consequence of low numbers of events, despite a meta-analysis of all existing studies.

Inconsistent capture or reporting of infections has an impact on the weighting of studies within a meta-analysis. Fewer events within a study result in an increased variance and thus a lower weighting. We therefore applied alternative weightings including total number of patients and estimated total person time, so studies with high numbers of patients but few infections would contribute more weight to the meta-analysis. For example, a 2-year study of 250 patients with one discontinuation for infection [33] contributed only 2.7% weight to the original meta-analysis, but increased to 17.6% when weighted by numbers of patients or 23.2% by person-time. The absence of a significantly increased risk in these sensitivity analyses is reassuring, although again, we cannot conclude that GCs are not associated with an increased (or decreased) risk of infection: the confidence intervals included up to a 70% increased or decreased risk, which is clinically meaningful.

### Heterogeneity in observational studies

Although RCTs have some heterogeneity, for example in background therapy or entry criteria, the variability in observational studies is much wider. The observational studies reflected a wide range of settings and populations, including year of recruitment, disease duration, disease severity, GC therapy practice, co-therapy, co-morbidity, geography, health-care systems, and recruitment methods (for example, single-center surgical experience, administrative database, biologics register). Each has its own implication for risk estimates, but the multiple domains of difference meant that much heterogeneity existed within the studies. Even after stratification within any chosen domain, many differences remained in the other areas of potential heterogeneity, and the I^2 ^values often remained high. Nonetheless, within this heterogeneity, the direction of effect typically suggested an increased risk associated with GC therapy, with only six of 42 studies reporting a relative risk of < 1. Statistical heterogeneity thus likely arose from different effect sizes.

It has been argued that meta-analysis of published nonexperimental data should be abandoned [84]. Others argue that careful consideration of sources of heterogeneity within a systematic review can offer more insights than the "mechanistic calculation of an overall measure of effect, which will often be biased" [85]. We ran many stratified analyses to consider the impact of these possible factors, producing some useful results, such as demonstrating a dose response.

### Lack of detailed reporting in observational studies

Clear reporting of methods and results was a problem in observational studies as well as in RCTs, in particular, the definition of GC exposure and methods of risk attribution. This is important for GC therapy in RA because of its intermittent pattern of use and multiple routes of administration. GC therapy was rarely the primary exposure of interest in these observational studies, but merely one of many possible exposures or covariates, perhaps explaining the lack of detail. Methods sections rarely reported clearly on how GC exposure was captured, although each study design provided certain opportunities for defining exposure. For example, in prescription databases, clinician reporting, or case note review without clarity about exposure, interpreting the many study results was challenging. Even when the source of exposure was clearly described, the definitions for "GC exposed" were rarely consistent. GC exposure was variously defined as ever exposed during the study period [37], exposed at study baseline [36], or recent [75] or current exposure [39] at the time of infection. Even within exposure categories, definitions varied. For example, current exposure at the time of infection included definitions of GC prescriptions within 30 days of the event, 45 days, and beyond. Risk windows used in the analyses included "on drug" [39,59], "on drug plus lag window" [68,71], and "ever exposed" [36,66]. Such analytic variability can produce different results even within one study [86]. Exploration of dose within observational studies was restricted by reporting. We were able to explore a possible dose-response only in studies that stratified by dose. Variability in the time period was found when average dose was considered, similar to yes/no definitions of exposure, adding additional heterogeneity. Definition and sources of outcomes as well as methods of verification (when undertaken) also varied between studies. Sources of infection ranged from electronic medical records, through case-note review or direct clinician reporting, to linkage with national inpatient registers.

Several risk estimates had to be excluded because of problems with reporting, including typographic errors with point estimates outside of confidence intervals, and absent confidence intervals around reported point estimates [39,87]. Other studies reported average GC dose for cohorts of patients, but the absence of absolute patient numbers receiving GC therapy prevented inclusion.

### Confounding and bias in observational studies

Confounding by disease severity, whereby patients with more-severe disease (and thus at a higher risk of infection) are more likely to receive steroids, was a major concern. This potential bias is unavoidable in observational drug studies. Confounding by contraindication was another possibility, in which patients with high comorbidity or frailty are considered too high risk for traditional DMARDs, and are instead treated with GCs. Within the meta-analysis, we stratified studies into those that reported unadjusted and adjusted risk estimates. Interestingly, the adjusted analyses provided a higher estimate of risk than did the unadjusted analyses, contrary to what we expected. If high disease severity and high comorbidity were reasons for receiving GC therapy (and both are independent risk factors for infection), we would have expected the adjusted analyses to move toward the null. However, clinical decisions are complex, and more than these two variables are considered, leaving the possibility of residual confounding.

Publication bias is an important consideration, present at several levels. First, researchers who found a positive "statistically significant" association between GC therapy and infection risk may be more inclined to include this result in their article. Indeed, 23 of 42 observational studies had statistically significant increased risks, with several just reaching the threshold of significance.

Second, techniques such as forward or backward selection for multivariate analysis automatically reject nonsignificant results. If GC therapy was only one of many covariates of interest, it is plausible that only the significant results were reported. We found examples of studies in which GC therapy was included in a multivariate model, but no subsequent GC risk estimate was reported [88]. At times, it was explicitly reported that no association was found, but either no measure of effect was provided [89-93], or only a *P *value > 0.05 was reported [94]. Exclusion of these null studies would result in a false inflation of the summary risk estimate and is a major concern.

Third, having discovered a significant association, researchers may be more inclined to submit for publication.

Fourth, reviewers may be more inclined to accept. Publication bias means that the infection risk with GC therapy is likely to be less than the estimated RR of 1.67. Unfortunately, we cannot know how far correction for publication bias would move the result toward the null.

### Quality of included studies

When combining multiple studies, we must consider not only the results from those individual studies, but also the quality of the studies. At present, no accepted instruments are available to assess the quality of studies that evaluate harms [82,95]. We did attempt to assess the included studies according to scales but found that the scores oversimplified the limitations, lacked discrimination between studies, and missed other important factors. For example, the McHarm scale [96] scores reporting of both serious and severe harms as well as deaths. Very few of the observational studies had the primary aim of examining the safety of GC therapy, and thus did not consider severity or death. The Newcastle Ottawa Scale [97] includes a domain about comparability, or adjustment for confounders. The majority of studies adjusted for confounders, yet wide variation existed in the covariates used. We have listed the confounders adjusted for within Table AF2 to provide the reader with study-specific details and performed sensitivity analyses by using different adjustments. Ascertainment of exposure and outcome [97], as already discussed, was challenging to assess because GC therapy was only one of many covariates and often not the primary exposure of interest. Such lack of detail meant that we were limited in generating a meaningful or accurate score. Nonetheless, no studies appeared to have different methods of ascertainment of the exposure/outcome for the cases and controls exposed and comparison cohorts.

## Conclusions

Given these numerous problems with both study designs in assessing the infection risk with GC therapy, how can we best summarize? The interventional nature of RCTs provides an opportunity to isolate and examine the effect of therapy. To overcome the problem of small numbers of events in individual studies, meta-analysis can collate results and enhance this useful experimental study design to address safety. Multiple analytic models all reached the same broad estimate, providing reassurance. Unfortunately, all estimates were derived from the selected studies after exclusion of studies of lower-quality methods and reporting. The results are valid only if the included studies were representative of all studies, and this is something we cannot assess. Of greater concern was the outcome ascertainment and reporting, which was generally of poor quality. The clear variation in methods of ascertainment and reporting within our included studies, plus likely underreporting, leads to anxiety about the meta-analysis result. The observational studies are harder still to untangle. Many issues cloud the picture, in particular methods for defining exposure and risk attribution, residual confounding, and publication bias. Replication of results does not allay these concerns. We must conclude that the risk of infection associated with systemic GC therapy in patients with RA remains uncertain, despite six decades of clinical experience. However, one consistent finding is that we cannot rule out the possibility of a clinically important increased risk, from either the RCTs or the observational studies. Improved, standardized reporting of harms [98] and improved access to patient-level, time-dependent data from RCTs would improve the ability to assess adequately the risks of specific adverse events. Within observational studies, clear definitions of drug exposure and risk attribution, as well as reporting of effect sizes, irrespective of statistical significance [99], would advance our knowledge.

## Abbreviations

aHR: adjusted hazard ratio; aRR: adjusted relative risk; CI: confidence interval; DMARD: disease-modifying antirheumatic drug; EARA: extra-articular RA; GC: glucocorticoid; HAQ: health-assessment questionnaire; HR: hazard ratio; IRR: incidence rate ratio; iv: intravenous; ivMP: intravenous methylprednisolone; LEF: leflunomide; MTX: methotrexate; n/a: not available; NDB: National Data Bank for Rheumatic Diseases; NSAIDs: nonsteroidal anti-inflammatory drugs; OR: odds ratio; PEQ: prednisolone equivalent; Pyrs: person years; RA: rheumatoid arthritis; RCT: randomized controlled trial; RhF: rheumatoid factor; RR: relative risk; SSZ: sulfasalazine; TB: tuberculosis; TNF: tumor necrosis factor.

## Competing interests

The authors declare that they have no competing interests.

## Authors' contributions

All authors jointly conceived the study. WGD generated the search strategy and performed the initial abstract screening. WGD and MH independently reviewed the 430 full-text articles. WGD performed the data extraction and meta-analysis. All authors helped to draft the manuscript and read and approved the final manuscript.

**Figure 4:**
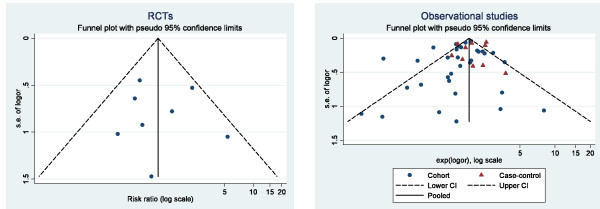
**Funnel plots of risk ratios in (a) RCTs and (b) observational studies, stratified by study design**.

## Supplementary Material

Additional file 1 **Search strategy for identifying RCTs and observational studies**. Click here for file

Additional file 2 **Observational studies reporting risk of infection outcomes by GC therapy**. Click here for file

Additional file 3 **Sensitivity analyses of RCT and observational study meta-analyses**. Click here for file
