# Supplementary material for: The association between systemic glucocorticoid therapy and the risk of infection in patients with rheumatoid arthritis: systematic review and meta-analyses
Source: Arthritis Res Ther. 2011 Aug 31;13(4):R139. doi: 10.1186/ar3453 (PMC3239382; doi:10.1186/ar3453)
Supplement: Additional file 1 — Search strategy for identifying RCTs and observational studies. [file ar3453-S1.DOC]

**Appendix A.** Search strategy

Ovid search terms across EMBASE and MEDLINE:

**[1]** **Infection:** exp Infection/ or infection.mp. or infection.m_titl or listeria.mp. or exp Listeria Infections/ or legionella.mp. or exp Legionella/ or coccidioidomycosis.mp. or exp Coccidioidomycosis/ or histoplasmosis.mp. or Histoplasmosis/ or zoster.mp. or exp Herpes Zoster/ or exp Tuberculosis/ or tuberculosis.mp. or xp Pneumocystis Infections/ or pneumocystis.mp. or pneumonia.mp. or exp pneumonia/ or septic arthritis.mp. or exp bacterial arthritis/ or cellulitis.mp. or exp cellulitis/ or pyelonephritis.mp. or exp acute pyelonephritis/ or osteomyelitis.mp. or exp osteomyelitis/

**[2]** **Anti-rheumatic therapy:** exp Glucocorticoids/ or glucocorticoids.mp. or steroid*.mp. or corticosteroid*.mp. or corticosteroid.m_titl. Or glucocorticoid.m_titl. Or exp Antirheumatic Agents/ or infliximab.mp. or adalimumab.mp. or etanercept.mp. or anti-TNF.m_titl. or anakinra.mp. or rituximab.mp. or abatacept.mp. or sulphasalazine.mp. or leflunomide.mp. or chloroquine.mp. or Hydroxychloroquine/ or Gold/ or Gold Compounds/ or gold.mp. or chloroquine.mp. or Chloroquine/ or methotrexate.mp. or Methotrexate/ or sulfasalazine.mp. or Sulfasalazine/ or DMARD.m_titl

**[3]** **RA:** rheumatoid arthritis.mp. or exp Arthritis, Rheumatoid/ or inflammatory polyarthritis.mp. or inflammatory arthritis.mp.

*Observational studies*

EMBASE

([1] AND [2] AND [3] AND (risk*.mp. or risk/ or cohort stud*.mp. or cohort analysis/ or case control study.mp. or case control study/ or incidence ratio.mp. or incidence density ratio.mp. or odds ratio.mp. or incidence rate ratio.mp. or rate ratio.mp.)) limited to (human and english) excluding (book or book series or conference paper or editorial or note or proceeding or "review" or short survey))

MEDLINE

([1] AND [2] AND [3] AND (risk*.mp. or risk/ or cohort stud*.mp. or cohort analysis/ or case control study.mp. or case control study/ or incidence ratio.mp. or incidence density ratio.mp. or odds ratio.mp. or incidence rate ratio.mp. or rate ratio.mp.)) limited to (human and english) excluding (addresses or bibliography or biography or case reports or clinical trial, phase i or clinical trial, phase ii or congresses or consensus development conference or consensus development conference, nih or dictionary or directory or duplicate publication or editorial or festschrift or government publications or guideline or in vitro or interactive tutorial or interview or lectures or legal cases or legislation or meta analysis or news or newspaper article or patient education handout or periodical index or portraits or practice guideline or retracted publication or "retraction of publication" or "review" or "scientific integrity review")

MEDLINE In-Process & Other Non-Indexed Citations

([1] AND [2] AND [3] AND (risk*.mp. or risk/ or cohort stud*.mp. or cohort analysis/ or case control study.mp. or case control study/ or incidence ratio.mp. or incidence density ratio.mp. or odds ratio.mp. or incidence rate ratio.mp. or rate ratio.mp.))

CINAHL (not limited to observational studies in search)

((“rheumatoid arthritis”) or (MH “Arthritis, Rheumatoid+”)) AND ((“infection”) or (MH “Infection+”) or (MH “Bacterial Infections”) or (“fungal infection”) or (MH “Mycoses+”) or (“viral infection”) or (MH “Virus Diseases+”)  or (“parasitic infection”) or (MH “Parasitic Diseases+”) or (“pneumonia”) or (MH “Pneumonia+”)  or (“septic arthritis”) or (MH “Arthritis, Infectious”) or (“osteomyelitis”) or (MH “Osteomyelitis”)  or (“cellulitis”) or (MH “Cellulitis”) or (MH “Fasciitis, Necrotizing”) or (“pyelonephritis”) or (MH “Pyelonephritis”)  or (“tuberculosis”) or (MH “Tuberculosis”) or (“zoster”) or (MH “Herpes Zoster”) or (“pneumocystis”) or (MH “Pneumonia, Pneumocystis Carinii”) or (“legionella”) or (MH “Legionella”) or (“listeria”) or (MH “Listeria Infections”) or (“coccidioidomycosis”) or (MH “Coccidioidomycosis”) or (“histoplasmosis”) or (MH “Histoplasmosis”)) AND ((“disease modifying anti-rheumatic drug”) or (MH “Antirheumatic Agents”) or (“glucocorticoids”) or (MH “Glucocorticoids”) or (“corticosteroid”) or (MH “Adrenal Cortex Hormones”) or (“steroid”) or (MH “Steroids”))

*Clinical trials*

EMBASE

((rheumatoid arthritis.mp. or exp Arthritis, Rheumatoid/ or rheumatoid arthritis.m_titl. or inflammatory arthritis.mp. or inflammatory polyarthritis.mp.) AND *Glucocorticoids/ AND exp clinical trial/) limited to (human and english)
