# Supplementary material for: The association between systemic glucocorticoid therapy and the risk of infection in patients with rheumatoid arthritis: systematic review and meta-analyses
Source: Arthritis Res Ther. 2011 Aug 31;13(4):R139. doi: 10.1186/ar3453 (PMC3239382; doi:10.1186/ar3453)
Supplement: Additional file 2 — Observational studies reporting risk of infection outcomes by GC therapy. [file ar3453-S2.DOCX]

**Table S1. Observational studies reporting risk of infection outcomes by GC therapy**

| **First author and year** | **Country** | **Setting & study size** | **Duration of study** | **GC exposure definition** | **Comparator** | **Type of outcome** | **Result** | **Adjusted for** |
| --- | --- | --- | --- | --- | --- | --- | --- | --- |
| Askling, 2007 [[35](#_ENREF_35)] | Sweden | Prospective national biologics register: 4167 anti-TNF treated patients | Recruitment 1999-2003. Mean follow-up 1.9 years | Baseline GC use (at start of anti-TNF treatment) | No GC use at baseline | Hospitalisation for infection | RR 0.90 (0.69 to 1.17) | Age, gender, RA disease duration, baseline DAS28, baseline HAQ, DMARD use, 4 co-morbidities |
| Bergstrom, 2004 [[37](#_ENREF_37)] | US | Single-centre retrospective cohort study: 985 patients, 845 with RA | Follow-up 3 years | Not clear | No GC use | Coccidiodomycosis | OR 1.23 (0.36, 4.25) | Univariate |
| Bernatsky, 2007 [[38](#_ENREF_38)] | Canada | Nested case-control study in provincial administrative database. 1970 serious infections from 23 733 RA patients | Study period 23 years | Current use, defined as dispensed prescription within 45 days of index date | No current GC use | Hospitalisation for infection | All-site infections: RR 2.56 (2.29, 2.85)  Pneumonia: RR 2.07 (2.37, 3.08) (sic) | Age- and gender-matched, other DMARDs/ biologics, number of physician visits. Adjustment for co-morbidity unclear |
| Bongartz, 2008 [[39](#_ENREF_39)] | US | Single centre retrospective study of 462 patients with RA undergoing hip or knee replacement | Recruitment over 8 years 1996-04. Mean follow-up 4.3 years. | Peri-operative GC use | No GC use | Prosthetic joint infection | HR 1.28 (0.46–3.60) | Univariate |
| Brassard, 2006 [[40](#_ENREF_40)] | US | Nested case-control study within PharMetrics administrative database. 386 cases of TB from 112,300 patients with RA | Study period 1998-2003 | Current exposure, defined as dispensed prescription within 30 days of index date | No current GC use | Tuberculosis | RR 1.7 (1.3, 2.2) | Age, sex, co-morbidity, DMARDs/ biologics, NSAID use as surrogate for disease severity |
| Brassard, 2009 [[41](#_ENREF_41)] | Canada | Nested case-control study in provincial administrative database. 50 cases of TB from 24,282 RA patients | Study period 1980-2003. TB incidence estimated 1992-2003 | Current exposure, defined as dispensed prescription within 30 days of index date | No current GC use | Tuberculosis | RR 2.4 (1.1–5.4) | Age, sex, co-morbidity, DMARDs/ biologics, NSAIDs, number of physician visits |
| Breedveld, 1987 [[42](#_ENREF_42)] | The Nether-lands | Cohort study of 46 patients with Felty’s syndrome | Study period 1982-85. Mean follow-up 2.4 person years | GC use during a three-month period | No GC use during three-month period | All-site infections (20 ‘major’, 95 ‘minor’) | Infection rates/ 100 patient quarters:  All infections: 24 for 0mg PEQ, 29 for 1-20mg PEQ, 88 for >20mg PEQ  Major: 5, 5, & 8/ 100 PQ for 0, 1-20 & >20 PEQ, respectively | n/a  (O:E ratio estimated, allowing for PMN lymphocyte and monocyte counts) |
| Carpenter, 1996 [[43](#_ENREF_43)] | US | Single centre prospective study of post-operative infections in 32 methotrexate-treated patients following elective joint surgery | Study period 1982-91. Follow-up for at least 1 year | GC use at time of surgery | No GC use at time of surgery | Infection within one month of surgery | 3/4 post-operative infections received GCs compared to 19/28 procedures without infection | Crude numbers |
| Coyne, 2007 [[44](#_ENREF_44)] | UK | Single centre study of 1522 patients with RA | 1 year | Not clear | No GC use | Hospital admission with acute LRTI | 14/36 cases, 219/1486 controls | Crude numbers |
| Curtis, 2007 [[45](#_ENREF_45)] | US | Retrospective cohort study of 5326 MTX or anti-TNF treated RA patients from administrative database | Median follow-up 17 months | Use in the 6 months preceding index date | No GC use in 6 months preceding index date | All-site hospitalised infections | <5mg HR 1.49 (0.82–2.72)  5-10mg 1.46 (0.84–2.54)  >10mg 1.85 (1.21–2.85) | Age, biologic therapy, insurance type, number of physician visits, comorbidity |
| Doran, 2002 [[46](#_ENREF_46)] | US | Cohort study of 609 patients with RA from single centre | Recruitment 1955-94. Mean follow-up 13 years | Considered as time-varying covariate:  ever/ never use | Never use | All-site infections requiring hospitalisation | HR 1.56 (1.20–2.04) | Age, gender, comorbidities, EARA, RhF. Other treatments rejected from multivariate model |
| Edwards, 2007 [[47](#_ENREF_47)] | UK | 34,250 patients with RA from a primary care database | Study period 1987-2002. Mean follow-up for RA cohort 5.3 years. | GC treatment two months prior to infection | No prednisolone or DMARDs | Septic arthritis | IRR prednisolone only vs no prednisolone or DMARD 2.94 (1.93–4.46) | Co-morbidity,  smoking status, and age category (not gender) |
| Favalli, 2009 [[48](#_ENREF_48)] | Italy | 1064 patients from a regional population-based registry of anti-TNF users. | Mean follow-up 1.9 person years. | ‘Concomitant treatment’ | No concomitant prednisolone | All-site serious infection | HR prednisolone 0-5mg 1.53 (0.69 3.38), prednisolone >5mg 2.69 (1.13 6.41) | Age, gender, disease severity (DAS28), disease duration, RhF, anti-TNF therapy, MTX, co-morbidity |
| Fleischmann, 2006 [[49](#_ENREF_49)] | Inter-national | Open label extension study of 1346 anakinra-treated patients with RA following 6 month RCT. | Up to 3 years follow-up | Baseline GC therapy | No baseline GC therapy | a) All-site serious infection  b) Serious lower respiratory tract infection | a) 27 infections in 940 pyrs (2.87/100 pyrs) in non-GC cohort and 95 infections in 1333 pyrs (7.13/100 pyrs) in GC cohort  b) 1.17 vs 2.11/100 pyrs (11 and 30 infections, respectively) | Not adjusted |
| Franklin, 2007 [[36](#_ENREF_36)] | UK | Inception cohort of 2108 patients with inflammatory polyarthritis | Recruitment 1990-99. Mean follow-up 8 years | Ever GC use | Never used GC therapy | Hospitalisation for all-site infection | RR 2.2 (1.5 to 3.4) | Age, gender, ACR RA criteria, RhF, smoking, DMARD use (ever/ never), erosions, HAQ |
| Giles, 2006 [[50](#_ENREF_50)] | US | 91 patients with RA who had ≥1 orthopaedic procedure | Study period 1999-2004. | GC use at last rheumatology clinic visit prior to index date | No GC use at last visit | Septic  arthritis, osteomyelitis, or deep-wound infection in an instrumented  bone or joint requiring intravenous antibiotics within 30 days of procedure | 3/10 (30%) patients with infection on GCs vs 36/81 (44%) without infection on GCs | Not adjusted |
| Grijalva, 2010 [[51](#_ENREF_51)] | US | Cohort study of 14 586 patients with RA from US administrative database | Study period 1995-2005. Patients followed for up to 180 days after new treatment start. | New episodes of GC use, categorised as <7.5 (low), 7.5–30 (medium) and >30mg PEQ (high) | New episodes of MTX use (+/- GC therapy) | a) Serious infections that required hospitalization  b) Pneumonia | a) Low HR 1.62 (0.94, 2.78), Medium 2.39 (1.63, 3.51), High 3.72 (2.37, 5.84)  b) Low HR 2.30 (1.2, 4.41), medium 2.36 (1.44, 3.87), high 4.33 (2.49, 7.54) | Adjusted for age, sex and propensity score (including co-morbidity and surrogates for disease severity) |
| Hamalainen, 1984 [[52](#_ENREF_52)] | Finland | Case control study of 136 post-operative wound infections following orthopaedic procedures | Study period 1975-8 | GC use at time of procedure | No GC use at time of procedure | Post-operative wound infection within 3 months of procedure | 52/136 infection cases on GCs, 44/136 matched controls | Not adjusted |
| Harigai, 2007 [[53](#_ENREF_53)] | Japan | Multicenter, case–control study of  pneumocystis pneumonia in patients with RA treated with infliximab | Not clear | Daily dose of prednisolone  of at least 6 mg. Unclear at what timepoint | Not clear | Clinical diagnosis plus supportive laboratory tests for *Pneumocystis*  *Jiroveci* pneumonia | 21 cases compared to 102 controls. HR 3.76 (1.37  to 10.3) | Not clear |
| Hernandez-Cruz, 1998 [[54](#_ENREF_54)] | Mexico | Nested case-control study of 195 patients with RA | Not clear | GC use, presumed at time of infection | No GC use, presumed at time of infection/ index date | Validated multi-site-specific infections | OR 1.6 (0.87, 2.93) | Unadjusted |
| Huscher, 2009 [[55](#_ENREF_55)] | Germany | Patient reported outcomes from 779 unselected RA patients from 9 centres | Events in 6 months prior to questionnaire | No GCs in past 12 months or GC therapy for >6 months (<5mg/day; 5-7.5mg/day; >7.5mg/day) | No GC use in preceding year | Mycosis | Mycosis seen in 4.5% no GC use, 5.8% of <5mg/day, 6.6% of 5-7.5mg/day and 8.2% of >7.5mg/day. Multivariate analysis not significant | Unadjusted |
| Jain, 2002 [[56](#_ENREF_56)] | UK | Single centre experience of 129 wrist surgery procedures in 80 RA patients over 5 year period | Mean follow-up 9 months (range 0.5-46) | 4 groups: MTX only; prednisolone only; MTX & prednisolone; neither GC or MTX | No GC or MTX | Post-operative wound infection | Post-operative infections in 1/60 GC users (0/30 pred only, 1/30 pred & MTX) vs 5/69 non-GC users (3/48 MTX only and 2/21 no drug)  RR 0.23 (.028, 1.91) | Crude numbers |
| Jenks, 2007 [[57](#_ENREF_57)] | New Zealand | Single centre case note audit of 171 leflunomide-treated patients with RA | Study period 2002-06. Mean treatment duration 2 years. | Not clear | No GC use | Inpatient hospitalisation for infection | 9/99 GC users developed infection; 2/72 non-GC users developed infection | Crude numbers |
| Lacaille, 2008 [[58](#_ENREF_58)] | Canada | Retrospective cohort  study of 27710 patients with RA using administrative database. | Follow-up from 1996-2003. Mean follow-up 3.6 years. | Current oral GC exposure with or without DMARDs (lapses of <4 months considered as continuous use) | No current GC or DMARD use | a) serious infection (requiring hospitalization  or occurring during the course of a hospitalization)  b) mild infection (requiring a  physician visit or use of antibiotic medications) | *GC without DMARDs*  a) aRR 1.9 (1.75, 2.05)  b) aRR 1.15 (1.11, 1.19)  *GC with DMARDs:*  a) aRR 1.63 (1.5, 1.77)  b) aRR 1.12 (1.08, 1.16) | Age, sex, RA disease duration, co-morbidity, SES, prior infection |
| Luessenhop, 1996 [[59](#_ENREF_59)] | US | Retrospective study of patients (54 with RA) who had multiple prostheses  with at least one prosthetic infection | Study period 1981-93. Average follow-up not stated. | GC use at the time of the index infection | No GC use at time of index infection | Infection of a second arthroplasty in patients with multiple arthroplasties and a prior prosthetic infection | 9/19 patients with second infection on GCs at index date compared to 15/35 with no further infection | Crude numbers |
| Malysheva, 2008 [[60](#_ENREF_60)] | Germany | Retrospective cohort study of 154 patients with RA | Mean follow-up 12.6 years (max 39 years) | Not clear | Not clear | Not defined | OR infection DMARD + GC vs DMARD alone 3.39 (0.44–20.1) | Adjusted for duration  of DMARD therapy, duration of low-dose GC therapy, and previous  type of DMARD therapy |
| McDonald, 2009 [[61](#_ENREF_61)] | US | Retrospective cohort study of 20,357 patients from Veterans Affairs administrative database | Study period 1998-2005. Mean follow-up 3.5 years | GC use at time of infection | No GC use at time of infection | Herpes zoster | aHR 1.41 (1.19–1.67) | Age, sex, DMARD and biologic therapy, co-morbidity |
| Mertz, 2007 [[62](#_ENREF_62)] | US | Retrospective cohort of 287 patients with RA from single centre | Study period 2000-2006. Average follow-up not clear. | GC use at time of event for those with infection, GC use at ANY TIME within study period for other patients | No GC use | Coccidiodomycosis | 7/9 (78%) cases treated with GCs compared to 199/278 (72%) non-cases | Crude numbers |
| Murata, 2006 [[63](#_ENREF_63)] | Japan | Retrospective review of 122 RA patients  with 214 elective orthopaedic procedures | Study period 2000-2003. | Daily dose of predisolone | n/a | Postoperative infection defined as reddening of wound, discharge from the wound, and/or  readministration of antibiotics occurring within 1 year after  surgery | OR 1.9 (1.0–3.9) (presumed per 1mg increase in prednisolone) | Not stated whether regression is univariate or multivariate |
| Saag, 1994 [[64](#_ENREF_64)] | US | Cohort study of 112 matched pairs with RA: chronic GC users (<=15mg/day for >1 year) matched to non-GC users (non-continuous or unexposed) | Mean study period 6.2 years | Categorical exposure defined at baseline. Yearly average and cumulative doses calculated. | Conditional logistic regression for matched pairs | Infection requiring hospitalization or surgical  intervention | OR for use of prednisolone 8.0 (1.0, 64.0) | Matched on age, sex and disease duration. Adjusted for bony erosions, RhF, EARA, prednisone dose, DMARD use,  baseline ESR, prior infections, cardiac/ endocrine/ ophthalmologic disease |
| Salliot, 2007 [[65](#_ENREF_65)] | France | Retrospective cohort study of 709 anti-TNF treated patients | Study period 1997-2004. Mean follow-up 1.5 years | Concomitant use of GCs at start of anti-TNF therapy | No concomitant GC use at start of anti-TNF therapy | Infectious events reported in medical files or during outpatient  Visits. | 157/245 (64%) patients with infections used GCs at baseline vs 258/464 (56%) without infections (p=0.03) | Univariate. GC use not significant in multivariate model |
| Schnabel, 1995 [[66](#_ENREF_66)] | Germany | Cohort study of 168 MTX-treated patients | Up to 30 months’ follow-up | Methylprednisolone use at the time of infection | No MP use at the time of infection | Infections requiring antibiotics, or herpes zoster | 30/65 infectious episodes in patients receiving MP. 133/185 patients at baseline using GCs | Crude numbers |
| Schneeweiss, 2007 [[67](#_ENREF_67)] | US | 15,597 DMARD-treated Medicare benificiaries aged 65 and older | Study period 1995-2003. Average treatment episode duration 4 months. | GC exposure defined as the days of active prescription plus 3 weeks beyond last supply | MTX initiators with no GC use at time of infection | Hospitalisation for a serious bacterial infection | aRR 2.14 (1.50–3.06). >5mg/day 1.34 (0.85–2.13)  ; 6-9mg/day 1.53 (0.95–2.48)  ; 10-19 mg/day 2.97 (1.89–4.68)  ; >20mg/day, 5.48 (3.29–9.11) | Adjusted for propensity score (including age, sex, co-morbidity, physician visits, EARA) |
| Sihvonen, 2006 [[68](#_ENREF_68)] | Finland | Cohort of 604 patients with prevalent RA from single centre | Recruitment 1988 with mortality assessed in 1999 | GC therapy categorised at baseline:  A) No GC (<1 month oral GC)  B) Oral GC for >1 month but <10 years  C) Oral GC use >10 years | Groups A, B & C compared. | Infection as the underlying or immediate cause of death | Underlying cause of death in  3/209 Group A, 2/276 Group B and 6/119 Group C.  Immediate cause of death in 12/209 Group A, 13/276 Group B and 15/119 Group C (p=0.013) | Not adjusted |
| Smitten, 2007 [[69](#_ENREF_69)] | US/ UK | Nested case-control analysis of 122,272 patients from US Pharmetrics claims database and 38,621 patients from UK General Practice Research Database (GPRD) | Study periods of 1998-2002 and 1990-2001, respectively. | Oral GC exposure at time of event | No DMARDs or oral GCs at time of event | Herpes zoster | aOR 2.51 (2.05–3.06) in PharMetrics database  aOR 1.46 (1.24–1.70) in GPRD | Age, sex, NSAIDs, comorbidities,  orthopedic procedures, number of health care & rheumatology visits |
| Smitten, 2008 [[70](#_ENREF_70)] | US | Retrospective cohort study  of 24,530 patients from PharMetrics database | Study period  1999 to 2006. Median follow-up 2 years | Oral GC use at the time of event | No oral GC use at time of event | Serious infection: a hospitalized  infection or an infection requiring outpatient parenteral antibiotics | aOR 1.92 (1.67–2.21) for any oral GCs. Dose-response seen: <5 mg/day: 1.32 (1.06-1.63), 6-10 mg/day: 1.94 (1.53-2.46), >10 mg/day: 2.98 (2.41-3.69) | Age, sex, other current RA medication use, comorbidities, orthopedic procedures, number of hospitalizations, and  rheumatologist visits. |
| Strangfeld, 2009 [[71](#_ENREF_71)] | Germany | National biologics register. Prospective cohort study of 5040 anti-TNF or DMARD treated patients | Recruitment 2001-06. Average follow-up 1.8 and 2.4 years for anti-TNF and DMARD-treated patients, respectively | GC use at follow-up | No GC use at follow-up | Physician-reported herpes zoster | aHR 1.86 (0.92-3.78) for 1-9mg PEQ; 2.52 (1.12-5.65) for ≥10mg PEQ | Age, sex, DAS28, disease duration, co-morbidity, DMARDs and anti-TNF therapy |
| Tanaka, 2003 [[72](#_ENREF_72)] | Japan | 82 leflunomide-treated patients undergoing joint replacement surgery. Randomised to discontinue LEF 2 weeks prior to surgery | 12 months | GC use at the time of surgery | No GC use at time of surgery | “Local infectious complications” | 5/5 patients with infections in LEF group (n=41) treated with GCs. 4/5 patients with infections in non-LEF groups treated with GC | Crude numbers |
| Wilson, 1990 [[73](#_ENREF_73)] | US | Nested case-control study of post-operative infection following knee arthroplasty in patients with RA | Study period 1973 to 1987 | Oral GC use | No oral GC use | Deep infection with positive bacterial culture from knee joint | GC therapy used in 34/45 patients with infection vs 55/90 matched patients without infection | Crude numbers |
| Wolfe, 2006 [[74](#_ENREF_74)] | US | 16,788 patients from National Data Bank for Rheumatic Diseases (NDB): Prospective cohort study of RA outcomes | Study period 2001-2004. Median follow-up time 2.5 years, mean 2.2 years. | GC use at the start of 6-month assessment period | No GC use at start of 6-month assessment period | Hospitalisation for pneumonia | aHR 1.7 (1.5–2.1)  ≤5mg PEQ:  1.4 (1.1-1.6)  5-10mg PEQ:  2.1 (1.7-2.7)  >10mg PEQ:  2.3 (1.6-3.2) | Age, sex, HAQ, comorbidity,  number of DMARDs or biologic agents, RA duration, smoking, education categories,  safety registry membership |
| Wolfe, 2004 [[75](#_ENREF_75)] | US | 6,460 infliximab-treated RA patients from NDB | Study period 2000-02. Mean follow-up 1.2 years. | GC use at the start of 6-month assessment period (presumed) | No GC use at start of 6-month assessment period (presumed) | Active tuberculosis receiving anti-TB therapy | 1 of the 4 TB cases in the INF-treated cohort treated with GC | Crude numbers |
| Wolfe, 2006 [[76](#_ENREF_76)] | US | 10,614 herpes-zoster naïve patients with RA from NDB | Study period 2001-05. Mean follow-up 2.8 years. | GC use in previous 6-month assessment period | No GC use in previous 6-month assessment period | Self-reported herpes zoster (80% validated) | aHR 1.5 (1.2–1.8) | Age, age  squared, sex, education level, HAQ, smoking, co-morbidity, other RA treatments |

n/a – not available

RR – relative risk, aRR – adjusted relative risk

HR – hazard ratio, aHR – adjusted hazard ratio

GC – glucocorticoids

TNF – tumour necrosis factor

PEQ – prednisolone equivalent

EARA – extra-articular RA

RhF – rheumatoid factor

IRR – incidence rate ratio

HAQ – Health assessment questionnaire (measure of disability)

MP – methylprednisolone

LEF – leflunomide

NDB – National Data Bank for Rheumatic Diseases

TB – tuberculosis

Pyrs – person years

OR – odds ratio
