# Supplementary material for: The association between systemic glucocorticoid therapy and the risk of infection in patients with rheumatoid arthritis: systematic review and meta-analyses
Source: Arthritis Res Ther. 2011 Aug 31;13(4):R139. doi: 10.1186/ar3453 (PMC3239382; doi:10.1186/ar3453)
Supplement: Additional file 3 — Sensitivity analyses of RCT and observational study meta-analyses. [file ar3453-S3.DOC]

**Appendix B. Sensitivity analyses**

*RCTs*

Alternative weighting by patient numbers and estimated person-time changed the RR estimate to 0.85 (0.41, 1.79) (Figure B1) and 0.68 (0.27, 1.71) (Figure B2), respectively.

Figure B1. Meta-analysis of RCTs of systemic GC therapy versus controls for infection, re-weighted by number of patients.

Figure B2. Meta-analysis of RCTs of systemic GC therapy versus controls for infection, re-weighted by person-moments of follow-up

The sensitivity analysis without continuity correction led only to a slight widening in the confidence intervals (RR 0.97 (0.69, 1.38)). Adding 0.5 events to each cell led to no real change in the RR (0.96 (0.69, 1.34)). The Mantel-Haenszel odds ratio with zero-cell correction and the inverse variance weighting using random effects generated a RR estimate of 0.89 (0.63, 1.24) for both methods. Where studies reported more than one infectious outcome, selection of the alternative outcome did not lead to any material change in the risk estimate (RR 0.98 (0.83, 1.15)).

There were too few studies reporting exclusively serious infections, and too few events in those studies, to warrant a robust meta-analysis. Studies considered to report predominantly non-serious infection generated a RR of 1.05 (0.89, 1.24).

Limiting analysis to those studies with a placebo comparator again did not change the risk estimate (RR 0.97 (0.69, 1.36)). Exclusion of studies reporting only withdrawals for adverse events again led to little change (RR 1.02 (0.72, 1.44)). Studies examining a constant dose of <10mg PEQ had a RR of 1.05 (0.29, 3.81). The wide confidence intervals are a reflection of the small number of studies (and consequently events) with this constant dose. Several studies had tapering doses, and were thus not included.

*Observational studies*

Meta-analysis of the unadjusted risk estimates generated a summary RR of 1.11 (0.72, 1.71) for cohort studies and 1.50 (1.06, 2.12) for case-control studies, whilst adjusted estimates led to a RR of 1.72 (1.49, 1.97) for cohort studies and 2.01 (1.62, 2.50) for case-control studies. Adjustment for age and sex led to a RR of 1.32 (0.97, 1.80) compared to no adjustment (1.78 (1.58, 2.01)). The RR after adjustment for direct measures of disease severity was not different from the estimate unadjusted for disease severity. Both were lower than the estimate from studies adjusted for surrogate measures of disease severity such as number of physician visits. Adjustment for co-morbidity and for other RA therapies (DMARDs and/or biologics) led to estimates ~40% higher than the unadjusted estimates.

Meta-analysis stratified by the definition of exposure led to varied estimates of risk. The lowest estimate was from studies defining GC exposure at baseline (RR 1.46 (0.87, 2.45)). Recent use was slightly higher (RR 1.56 (1.24, 1.96)) with current use higher still (RR 1.70 (1.47, 1.97)). Ever use was associated with a RR of 1.80 (1.29, 2.51), although one of the two included studies allowed GC exposure to post-date infection. The highest risk was seen where the definition of GC exposure was not clear (RR 2.35 (1.27, 4.36)).
